# Efficacy and safety of FangJiHuangQi granule in patients with heart failure: a protocol of randomized, placebo-controlled trial

**DOI:** 10.3389/fcvm.2026.1723249

**Published:** 2026-05-29

**Authors:** Deng Pan, Lu Liang, Qian Zhao, Jianwu Zheng, Houyong Zhu, Yu Wang, Cheng Bao, Tielong Chen

**Affiliations:** Department of Cardiology, Hangzhou TCM Hospital Affiliated to Zhejiang Chinese Medical University, Hangzhou, Zhejiang, China

**Keywords:** clinical trial, fangJiHuangQi granule, heart failure, protocol, traditional Chinese medicine

## Abstract

**Background:**

FangJiHuangQi Granules (FJHQG) is a Chinese patent medicine that alleviates heart failure (HF) symptoms derived from the formula FangJiHuangQi decoction. Previous experiment has revealed that FangJiHuangQi decoction alleviated pathological injury in rats with HF. This trial intends to evaluate FJHQG's efficacy and safety as a complementary therapy in addition to conventional drug treatment.

**Methods:**

This trial is a randomized, double-blind, placebo-controlled study that recruits 100 patients with HF. Participants will be randomly assigned into the intervention and control groups (*n* = 50 for each group). For a duration of 12 weeks, FJHQG will be administered to the intervention group, and the control group will receive standard therapy plus placebo. The primary outcome of the trial is the changes of six-minute walk test at week 24. Secondary outcomes include BNP levels, NYHA classification, major adverse cardiovascular events (MACE), and KCCQ quality of life scales. Other exploratory assessments at the beginning and after a 12-week period will include fluid balance, symptom score scales, echocardiograms, diuretic consumption, and biochemical indices. Monitoring of MACE will continue until 12 weeks after drug discontinuation.

**Discussion:**

This study will evaluate the efficacy and safety of FJHQG in HF treatment and to examine potential mechanisms, offering new insights into HF treatment.

**Clinical Trial Registration:**

ChiCTR.org, ChiCTR2400080029.

## 1 Introduction

Heart failure (HF) is a complicated medical disorder triggered by multiple elements that cause detrimental modifications in cardiac structure and functionality. HF affects more than 64 million people worldwide (1). In industrialized countries, heart failure typically affects about 1% to 2% of adults, causing a heavy symptom and economic burden worldwide (2). Consequently, there is an immediate need to develop new drugs to reduce clinical event risks in patients with HF.

HF is often treated with Traditional Chinese Medicine (TCM) because of proven efficacy in ameliorating symptoms. According to TCM theory, HF mainly arises from a deficiency of heart Qi and blood stagnation. This condition denotes a pathological state wherein Qi is unable to facilitate effective blood circulation, leading to impaired or stagnant blood flow. Clinically, heart Qi deficiency usually manifests as fatigue, shortness of breath, palpitations, and impaired exercise tolerance, reflecting insufficient cardiac driving force. Blood stasis is associated with chest tightness or pain, and cyanosis of the lips, indicating impaired blood circulation. Water stasis results from an inability of Qi deficiency and blood stasis to regulate fluid metabolism and is typically manifested by peripheral edema, orthopnea, abdominal distension, and reduced urine output, which are typical symptoms of HF. The FangJi HuangQi (FJHQ) decoction, a renowned prescription in TCM comprises the following Chinese medicinal ingredients: *Astragalus membranaceus* (Fisch.) Bge. (6 g), *Stephania tetrandra* S. Moore (1.5 g), *Atractylodes macrocephala* Koidz*.* (3 g), *Glycyrrhiza uralensis* Fisch. (1 g), *Lepidium apetalum* Willd. (2.5 g), *Prunella vulgaris* L. (3 g), and *Salvia miltiorrhiza* Bunge (4 g). This formulation is reputed for its ability to “reinforce qi and improve blood circulation, invigorate spleen function, and clear water”. Extensive preliminary research has demonstrated that the herbs in the FJHQ decoction are both safe and effective (3, 4). Moreover, toxicological evaluations in rats revealed no noticeable negative impacts on the central nervous, respiratory, or cardiovascular systems, thus confirming the compound's safety (5, 6). In rats with pulmonary artery hypertension, the FJHQ decoction improved hemodynamic parameters, enhanced lung function, and lessened pulmonary vascular remodeling (7). In addition, FJHQ decoction has shown its effects in several cardiovascular conditions including hypertension, myocardial ischemia, and cardiac fibrosis (8). It has been shown that FJHQ decoction effectively improved exercise capacity and reduced diuretic usage and resistance without significant liver or kidney damage or adverse events in small-scale clinical observations (9, 10). However, there is still a lack of clinical evidence regarding the effectiveness and safety of FJHQ decoction in patients with HF.

FJHQ decoction has been applied in clinical practice for over a thousand years. However, the preparation of traditional decoctions is often time-consuming and complicated. Compared with traditional decoctions, the granule formulation provides improved quality consistency, enhanced reproducibility, and better patient compliance due to its convenience, making it more suitable for use in randomized controlled trials. Hence, this study will assess the efficacy and safety of FangJiHuangQi granule (derived from the FJHQ decoction, with the same ingredient and proportion, FJHQG) in patients with HF. Moreover, we plan to explore the potential mechanisms of FJHQG in patients with HF.

## 2 Methods/design

### 2.1 Study design

This trial is a part of the project “Theoretical Research on Treating HF with TCM Compatibility Formula - Topic Five Group Distribution Combination Theory Research” (2018 YFC1704505), led by Tianjin University of Traditional Chinese Medicine. The Hangzhou Hospital of Traditional Chinese Medicine, acting as the appointed clinical trial unit, hosts the clinical trial. The investigation will be conducted in accordance with Good Clinical Practice standards and the ethical principles of the Helsinki Declaration (11, 12). The research will follow the SPIRIT guidelines for interventional trials (13). Additionally, it will strictly adhere to the guidelines set forth by the 2017 CONSORT-CHM Formulas for Chinese Herbal Medicine (14).

**Figure 1:**
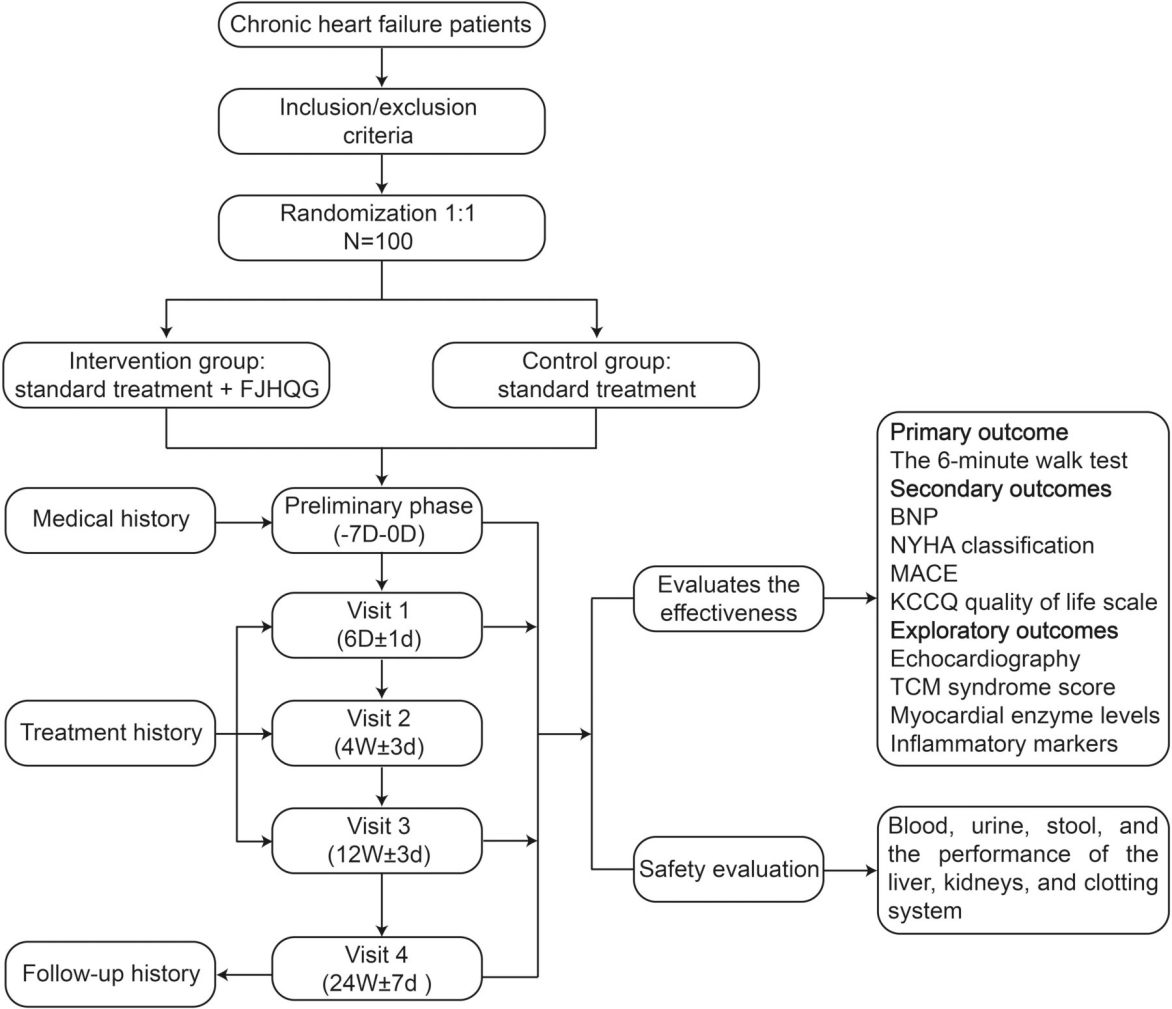
Flow diagram of the study.

This is a randomized, double-blind, placebo-controlled trial. The study will take place at Hangzhou Hospital of TCM and will include two stages: a 12-week treatment phase and a subsequent 12-week follow-up phase after intervention. A total of 100 patients with HF will be randomly assigned to two groups equally. On the basis of conventional drug therapy, the intervention group will receive treatment with FJHQG, while the placebo group will receive the corresponding placebo of FJHQG. Figure 1 depicts the study design.

### 2.2 Diagnostic criteria

The criteria for diagnosing HF follow the “2018 Chinese Guidelines for Heart Failure Diagnosis and Treatment Guideline” (15). The diagnostic criteria were defined according to the guidelines in effect at the time of study initiation and ethical approval.

The diagnosis of the TCM syndrome Qi deficiency, blood stasis, and water retention was based on the Technical Guidelines for Clinical Research of Traditional Chinese Medicine New Drugs in Chronic Heart Failure.

1. Qi deficiency and blood stasis syndrome

Major symptoms include: (1) shortness of breath or dyspnea; (2) fatigue; (3) palpitations. Secondary symptoms included: (1) general weakness and reluctance to speak; (2) spontaneous sweating; (3) low voice; (4) dark or purplish complexion or lips. Tongue and pulse signs included a dark tongue (with or without petechiae or ecchymosis, or engorged sublingual veins), thin white coating, and a deep, thready, uneven pulse or weak pulse. Diagnosis is established when at least two major symptoms and two secondary symptoms are present, combined with tongue and pulse manifestations.

2. Water retention syndrome

Symptoms include: (1) cough or sputum; (2) chest fullness or abdominal distension; (3) facial or limb edema; (4) oliguria. Tongue and pulse signs included a moist or greasy tongue coating or a slippery pulse. Diagnosis is established when at least one symptom is present, combined with tongue and pulse manifestations.

### 2.3 Inclusion criteria

The study will include participants who satisfy all of the following inclusion criteria:

1. Diagnosis of chronic heart failure in a stable condition (defined as no acute decompensation, and no hospitalization for heart failure within at least 2 weeks before randomization), classified as heart failure with mildly reduced ejection fraction (LVEF: 41%–49%) or heart failure with preserved ejection fraction (LVEF≥50%), with BNP ≥35 pg/mL, and at least one of the following echocardiographic features present: left ventricular hypertrophy and/or left atrial enlargement; diastolic dysfunction, according to the 2018 Chinese Guidelines for the Diagnosis and Treatment of Heart Failure, and meeting the criteria of “Qi deficiency, blood stasis, and water stasis” syndrome;
2. Patients must be on stable guideline-directed medical therapy for heart failure prior to randomization, defined as no major changes in medications within at least 2 weeks;
3. NYHA classification of II to III;
4. Age between 18 and 80 years;
5. Voluntary acceptance the medication treatment and provision of an informed consent form trained investigators.

### 2.4 Exclusion criteria

Participants will be excluded if they meet any of the exclusion criteria:

1. Unmanaged high blood pressure, defined by a resting systolic pressure of 180 mmHg or more, or a diastolic pressure of 110 mmHg or above, confirmed by two separate measurements before randomization;
2. Presence of acute coronary syndrome, recent or planned vascular revascularization within the past month or upcoming week, cardiogenic shock, life-threatening arrhythmias, myocarditis, constrictive pericarditis, pulmonary embolism, or other similar conditions;
3. Autoimmune conditions, including systemic lupus erythematosus and giant cell myocarditis;
4. Life-threatening conditions, including malignant tumors, or poorly controlled mental illnesses, as well as drug abuse;
5. Severe liver or kidney impairment, characterized by alanine aminotransferase levels at least three times the normal maximum or creatinine levels of 3 mg/dL or higher;
6. Known allergy to the medication being studied or any of its ingredients is known.
7. Women who are planning to become pregnant, are currently pregnant, breastfeeding, or not utilizing effective contraception during the trial period;
8. Involvement in additional clinical studies during the designated period.

### 2.5 Withdrawal, dropout, and discontinuation

The study will allow participants to withdraw at any point if: (1) the intervention threatens participants' safety; (2) a significant adverse reaction is observed; (3) the participant does not comply with the protocol. The reason for withdrawal will be documented in detail.

### 2.6 Randomization and blinding

This is a single-center study conducted at Hangzhou Hospital of TCM. Randomization will be performed by an independent statistician who is not involved in patient recruitment, intervention, or outcome assessment. The randomization sequence will be generated using SAS software version 9.4, with a 1:1 allocation ratio, stratified by sex, and age, utilizing random block sizes of six. Allocation concealment will be ensured using a centralized system, and researchers, participants, and data administrators and statisticians will be unaware of the randomization and treatment allocations. To ensure the blinding of participants and investigators, the placebo is identical to FJHQG in appearance, color, taste, packaging. Emergency unblinding will only be performed when necessary for patient safety and must be approved by principal investigator, and all such events will be documented.

### 2.7 Intervention

Every participant will be treated following the “2018 Chinese Guidelines for Heart Failure Diagnosis and Management” (15). This therapeutic plan involves diuretics, angiotensin-converting enzyme inhibitor (ACEI), angiotensin receptor blocker (ARB), angiotensin receptor-neprilysin inhibitor (ARNI), beta-blockers, and other suggested treatments. Optimal medical management will be ensured in alignment with the guidelines. All background medication will follow guideline-directed medical therapy. All concomitant medications and treatment adjustments during the study will be recorded in detail, and potential between-group differences will be summarized and adjusted. In addition, the intervention group will be given FJHQG, while the control group will undergo conventional drug therapy and corresponding placebo, both for a duration of 12 weeks. FJHQG is manufactured by CHIATAI QINGCHUNBAO PHARMACEUTICAL Co., Ltd. in accordance with Good Manufacturing Practice standards. All study medications will be provided by the manufacturer in standardized packaging. To ensure traceability, batch numbers will also be recorded (batch number: 2308702). The placebo will be identical to FJHQG in appearance, taste, and packaging to maintain blinding. Participants will be instructed to consume the granules (5 g per dose, twice daily). To guarantee adherence, the granules will be handed out and/or gathered during every visit. Adherence will be assessed based on sachet counts and patient-reported intake and will be calculated as the proportion of sachets taken relative to the total number prescribed. Missed doses and treatment interruptions will be recorded, and adherence below 80% will be considered a protocol deviation. During the trial, the use of other traditional Chinese medicine that may affect diuretic function is strictly prohibited.

### 2.8 Follow-up visits and monitoring schedule

The study will start with a 7-day preliminary phase where eligible individuals will provide their consent and initial information will be gathered. Future appointments will be planned for 6 days ± 1 day (visit 1), 4 weeks ± 3 days (visit 2), and 12 weeks ± 3 days (visit 3) after starting the treatment. Follow-up assessments will be conducted at Week 12 ± 7 days post-discontinuation of treatment (24W ± 7d) (visit 4). To minimize loss to follow-up, participants will be regularly contacted through telephone calls and WeChat group communication. Reminders will be provided before each scheduled visit to improve retention and adherence. Table 1 outlines the comprehensive assessment timetable.

**Table 1:** Schedule of enrollment, interventions, and assessments.

Study phase time	Lead-in period	Treatment period			Follow-up period
	Screening (−7D-0D)	Baseline (6D ± 1d)	Week 4 (4W ± 3d)	Week 12 (12W ± 3d)	Week 24 (24W ± 7D)^a^
Patients					
Eligibility screen	X				
Inclusion/exclusion criteria	X				
Informed consent	X				
General information	X				
Medical history	X				
Allocation	X				
Pregnancy examination	X				
Outcomes					
Primary outcome					
6-minute walk test	X	X	X	X	X^c^
Secondary outcomes					
BNP	X	X	X	X	X
MACE (including cardiac death, non-fatal myocardial infarction, and rehospitalization due to heart failure) ^b^	X	X	X	X	X
NYHA classification	X	X	X	X	X
KCCQ quality of life score	X	X	X	X	X
Exploratory outcomes					
Diuretic usage		X	X	X	X
Echocardiography	X			X	X
Heart failure-related rehospitalization rate^b^	X	X	X	X	X
TCM syndrome score	X	X	X	X	X
Myocardial enzyme levels	X			X	X
Inflammatory markers and other biochemical indicators	X			X	X
Safety					
Vital signs	X	X	X	X	X
Biochemical indexes	X			X	X
Electrocardiogram results	X			X	X
Adverse events	X	X	X	X	X
Others					
Drug distribution	X		X		
Drug recycle			X	X	
Combined medication			X	X	
Confirmation of medication compliance		X	X	X	X

a Visit 4 corresponds to the follow-up assessment conducted at Week 24, which is 12 weeks after completion of the 12-week treatment period, as stated in manuscript.

b Continuous monitoring throughout the study.

c Primary endpoint assessed at week 24.

Basic details encompass full name, birthdate, sex, race, job status, and more. The patient's medical background encompasses a present diagnosis of HF and previous health records. Biochemical indicators encompass blood, urine, feces, and the functioning of the liver, kidneys, and coagulation system.

### 2.9 Outcomes

The primary outcome of the trial will be the variation in the 6-minute walk test by week 24 as compared to baseline. The assessment of 6MWT will be performed in accordance with standardized procedures. The test will be conducted in a 30-meter corridor, and standardized instructions and encouragement will be provided to all participants. To ensure patient safety and consistency, predefined stopping criteria including intolerable dyspnea, chest pain, dizziness, or any other symptoms indicating potential risk, as judged by the assessor, will also be applied. The use of walking aids or supplemental oxygen will be permitted if clinically necessary, and the use will be recorded to minimize variability. Moreover, all outcome assessors will remain blinded to treatment allocation throughout the study to reduce measurement bias. The same assessor will evaluate a given participant across study visits to reduce inter-rater variability. All assessors will receive standardized training prior to study initiation. The 6MWT will be performed 1–2 h after routine medication administration to minimize variability. Duplicate tests will not be performed unless clinically indicated.

The secondary outcomes will include: (1) major adverse cardiovascular events (MACE, including cardiac death, non-fatal myocardial infarction, and rehospitalization due to HF) at 3 and 6 months. All major adverse cardiovascular events and heart failure-related rehospitalizations will be reviewed and adjudicated by an independent committee blinded to treatment allocation; (2) BNP levels at week 24 compared with baseline; (3) NYHA class functional classification at week 24 compared with baseline; (4) quality of life score of the Kansas City Cardiomyopathy Questionnaire at week 24 compared with baseline.

Other exploratory outcomes will include: (1) clinical outcomes including diuretic dosage, and 24-hour fluid intake and output (recorded for inpatients only); (2) TCM syndrome scale scoring; (3) cardiac structure outcomes including left ventricular ejection fraction, diastolic diameter, interventricular septal thickness, and posterior wall thickness indicated by echocardiography. Image acquisition and measurements will follow predefined procedures, and all echocardiographic data will be interpreted by experienced cardiologists who are blinded to treatment allocation to minimize assessment bias; (4) biochemical measurement outcomes including lipid profile (including total cholesterol, triglycerides, high-density lipoprotein cholesterol, low-density lipoprotein cholesterol, apolipoprotein A1, apolipoprotein B, and homocysteine), myocardial enzymes [including lactic dehydrogenase, creatine kinase (CK), and CK-MB], inflammation indicators [including C-reactive protein (CRP) and interleukin-6 (IL-6)] and other biochemical parameters (including bioactive adrenomedullin, CD146, vasopressin 2 receptor, cyclic adenosine monophosphate, protein kinase A, aquaporin 2, mucin 16, stromelysin 2, and soluble stromelysin 2). Specifically, bioactive adrenomedullin and CD146 are associated with endothelial function and vascular integrity, reflecting microcirculatory dysfunction. Vasopressin 2 receptor and aquaporin 2 are involved in water metabolism and fluid retention, representing fluid imbalance. Cyclic adenosine monophosphate and protein kinase A are key components of intracellular signaling pathways related to neurohormonal activation. In addition, stromelysin 2, soluble stromelysin 2, and mucin 16 are associated with extracellular matrix remodeling, inflammation, and tissue injury. Together, these biomarkers provide a comprehensive evaluation of endothelial dysfunction, fluid regulation, and neurohormonal activation, which are closely related to heart failure progression and may potentially reveal the mechanism of FJHQG in treatment of HF. Blood plasma will be gathered and kept at −80 °C in the lab freezer for analysis. Exploratory outcomes will be interpreted descriptively and will not be used for confirmatory inference.

### 2.10 Safety assessment

Safety evaluations will be performed during Visits 1, 3, and 4, including standard tests of blood, urine, stool, and functions of the liver, kidneys, and coagulation system. Laboratory abnormalities will be evaluated based on severity grading and clinical significance. The drug will be discontinued in the event of serious adverse events, intolerable adverse reactions, or clinically significant abnormalities in liver, renal, or hematologic parameters, as judged by the investigator.

### 2.11 Adverse events

In the course of the trial, adverse events (AEs) will be defined as unforeseen or harmful clinical situations. In the event of such occurrences, patients will receive immediate intervention. The ethics committee will receive comprehensive reports on all aspects of AEs within 24 h. The relationship between AEs and the study drug will be assessed as definitely related, possibly related, possibly unrelated, definitely unrelated, or indeterminate.

### 2.12 Data collection and management

Data will be initially recorded using paper-based case report forms (CRFs). All CRFs will be subsequently entered into an electronic data management system by independent data administrators. Double data entry and cross-validation will be also performed to ensure data accuracy and completeness. Original paper CRFs will be securely archived for verification. Methods including double data entry, data cleaning, manual verification, and additional validation techniques will be used to verify the data's validity, accuracy, and completeness. Due to the relatively small sample size, a formal Data and Safety Monitoring Board will not be established. Nonetheless, participant safety will be monitored throughout the study by the principal investigators and the institutional ethics committee, with all adverse events reviewed and reported in accordance with Good Clinical Practice guidelines.

### 2.13 Researcher education and assurance of quality

Researchers will receive a prepared Standard Operating Procedure (SOP). Every researcher will receive training to guarantee uniformity. The program encompasses evaluating qualified participants, employing survey instruments, among other activities. The WeChat group will aim to foster connection among patients. To maintain high standards, the quality control team and monitors from Hangzhou Hospital of TCM will conduct frequent inspections.

A protocol will be created for researchers at every facility to guarantee uniformity and standardization. Researchers will complete detailed training that covers screening eligible participants, administering survey scales, performing the 6-minute walk test, and other methods. A WeChat group will also be utilized to facilitate close communication with patients. The quality control team and inspectors designated by Hangzhou Hospital of TCM will carry out routine oversight. Monitoring activities will be implemented to ensure adherence to the study protocol and data integrity. The study team will conduct regular reviews of informed consent documentation, case report forms, and study drug accountability. Participant safety will be monitored throughout the trial, with adverse events evaluated and recorded at each visit.

To ensure the quality and consistency of the trial, roles and responsibilities of researchers are clearly assigned. Investigators are responsible for patient recruitment, clinical assessment, intervention implementation, and outcome evaluation. Data administrators independently conduct data entry, verification, and management. Clinical monitors perform regular on-site monitoring visits according to SOP to ensure adherence to the study protocol, accuracy of case report forms, and completeness of source data documentation. Monitoring activities include verification of informed consent, drug accountability, and outcome assessments. In addition, all clinical laboratory assessments are conducted using standardized procedures in certified laboratories to ensure consistency and reliability of results.

### 2.14 Sample size calculation

Patients will be randomized in a 1:1 ratio to receive either FJHQG or placebo. Previous studies in HF suggested that improvements of approximately 30 meters in the 6MWT is clinically meaningful. According to our previous clinical observation, assuming FJHQG demonstrates an improvement of 50 meters in 6MWT compared with placebo and a standard deviation of 72 meters among two groups, a two-sided significance level of *α* = 0.05, and a statistical power of 90%. The results indicate that at least 45 patients will be needed per group. Considering an anticipated dropout rate of 10%, a total of 100 patients will be enrolled, with 50 allocated to the intervention group and 50 to the placebo group. The study is powered for the primary functional endpoint and is not designed to detect differences in clinical events.

### 2.15 Statistical analysis

All statistical analyses will be conducted on both the intention-to-treat (ITT) and per-protocol (PP) populations. The ITT population will include all randomized participants, while the PP population will consist of participants with at least 80% adherence to the protocol. Continuous variables following a normal distribution will be presented as means ± standard deviations (SD) with 95% confidence intervals (CI), and non-normally distributed variables will be reported as medians with interquartile ranges (IQR) and analyzed using non-parametric methods. Between-group comparisons for normally distributed continuous variables will use the independent samples t-test, and for non-normal distributions, the Wilcoxon rank-sum test. Within-group comparisons will employ paired t-tests or Wilcoxon signed-rank tests as appropriate. Categorical variables will be summarized as counts and percentages, with between-group comparisons using the Chi-square or Fisher's exact test, and within-group comparisons using the McNemar test. Baseline characteristics will be reported descriptively without significance testing to assess comparability between groups. Differences in background treatment between groups will be summarized descriptively and adjusted for in the statistical analysis to reduce potential confounding.

The primary outcome (6-minute walk test) will be analyzed using analysis of covariance (ANCOVA) with baseline values and key covariates (age, sex) as covariates to adjust for potential confounding. Secondary outcomes, due to their diverse nature, will be analyzed using appropriate methods: continuous variables with t-test or Wilcoxon test, categorical variables with Chi-square or Fisher's exact test, and repeated measures or longitudinal data using linear mixed-effects models. To control for multiple comparisons among secondary outcomes, the false discovery rate (FDR) adjustment using the Benjamini-Hochberg method will be applied, and a hierarchical structure will be applied with the primary outcome taking precedence over secondary and exploratory outcomes. The primary analysis will be conducted according to the intention-to-treat (ITT) principle, including all randomized participants. If the proportion of missing 6MWT data is 5% or greater, missing data for the 6MWT will be handled using multiple imputation under the missing-at-random assumption. The imputation model will include baseline 6MWT value, age, sex, and treatment group as predictors. Imputation will be performed using a single model based on all available data without separating treatment arm. In addition, reasons for missing data will also be recorded, categorized, and summarized descriptively, and will be compared between groups. Prespecified subgroup analyses will be conducted based on heart failure phenotype, defined by left ventricular ejection fraction (LVEF ≥50% vs. <50%), and by sex. These subgroup analyses will be considered exploratory. Sensitivity analyses will be performed, including per-protocol analyses and complete-case analyses, to assess the robustness of the results. Deaths occurring before the final assessment will be handled in sensitivity analyses using a worst-case approach by assigning a value of zero for the 6MWT, representing the worst functional outcome. Adverse events will be summarized in tables and compared between groups using descriptive statistics. All analyses will be performed using SAS software version 9.4, and two-tailed *P*-values < 0.05 will be considered statistically significant. A detailed statistical analysis plan will be finalized prior to database lock to ensure prespecified analysis procedures.

## 3 Discussion

According to guideline recommendations, the primary treatment for HF includes ACEIs/ARNIs, sodium-dependent glucose transporters 2 inhibitors, beta-blockers, and mineralocorticoid receptor antagonist, etc (16). However, research indicates that the mortality rate for HF patients within one month of discharge, even after receiving conventional drug therapy remains high (17). This trial a randomized, double-blind, placebo-controlled, parallel-group trial that aims to assess the safety and clinical efficacy of FJHQG as an adjunctive treatment for HF. This trial is designed to provide preliminary clinical evidence supporting the potential application of FJHQG in HF treatment.

It has been demonstrated that FJHQ decoction has various pharmacological effects in different diseases (8). Regarding cardiovascular disease, FJHQ decoction has shown its effects in improving cardiac function in patients with HF. It has been demonstrated that FJHQ decoction improved ejection fraction, increased 24-hour urine production, reduced NT-proBNP levels, and relieved symptoms of HF (9, 10). In addition, we previously conducted a clinical trial with 74 patients suffering from HF with preserved ejection fraction and found that FJHQ decoction significantly reduced symptoms like shortness of breath and fatigue. Additionally, FJHQ decoction also improved the results of the 6-minute walk test and reduced serum BNP levels, the mechanism of which was associated with the reduction in serum sST2 and galectin-3 levels by FJHQ decoction (18). Moreover, it has also been found that FJHQ decoction is effective in improving renal function and increasing renal blood flow, which is also correlated with amelioration of cardiac function (19). Network pharmacology analyses have suggested that the potential effects of FJHQ decoction may involve modulation of neurohumoral signaling, vascular function, and myocardial stress–related pathways relevant to heart failure pathophysiology (20). Collectively, these findings provide a clinical and mechanistic rationale for the present randomized controlled trial.

Several limitations should be noticed in this trial. Firstly, the limited timeframe of the study will result in a short follow-up period for this trial. Long-term efficacy of FJHQG requires extended follow-up. Secondly, the current study will exclude participants with HF aged over 80, raising concerns about whether the results will apply to those over 80 years of age. Thirdly, the assumed effect size may be relatively optimistic and should be interpreted in the context of an exploratory study design. Finally, our study population will consist predominantly of Han Chinese individuals from southern China. Therefore, it will remain unclear whether the results can be generalized to different ethnic and regional groups.

In conclusion, this research will assess the efficacy and safety of FJHQG in patients with HF and will explore the fundamental mechanisms, providing fresh perspectives on HF treatment.

## References

[1] GBD 2017 Disease and Injury Incidence and Prevalence Collaborators. Global, regional, and national incidence, prevalence, and years lived with disability for 354 diseases and injuries for 195 countries and territories, 1990–2017: a systematic analysis for the global burden of disease study 2017. Lancet. (2018) 392(10159):1789–858. 10.1016/S0140-6736(18)32279-7 30496104 PMC6227754

[2] Groenewegen A Rutten FH Mosterd A Hoes AW. Epidemiology of heart failure. Eur J Heart Fail. (2020) 22(8):1342–56. 10.1002/ejhf.1858 32483830 PMC7540043

[3] Pan J Cao Z Fang C Lei Y Sun J Huang X. Huangqi shengmai yin ameliorates myocardial fibrosis by activating Sirtuin3 and inhibiting TGF-*β*/smad pathway. Front Pharmacol. (2021) 12:722530. 10.3389/fphar.2021.722530 34483934 PMC8414644

[4] Liu Y Xu W Xiong Y Du G Qin X. Evaluations of the effect of HuangQi against heart failure based on comprehensive echocardiography index and metabonomics. Phytomedicine. (2018) 50:205–12. 10.1016/j.phymed.2018.04.027 30466980

[5] Chu S Lu Y Liu W Ma X Peng J Wang X. Ursolic acid alleviates tetrandrine-induced hepatotoxicity by competitively binding to the substrate-binding site of glutathione S-transferases. Phytomedicine. (2022) 104:154325. 10.1016/j.phymed.2022.154325 35820303

[6] Dai Y Jin R Verpoorte R Lam W Cheng YC Xiao Y. Natural deep eutectic characteristics of honey improve the bioactivity and safety of traditional medicines. J Ethnopharmacol. (2020) 250:112460. 10.1016/j.jep.2019.112460 31837415

[7] Xue Z Zhou M Liu Y Qin H Li Y Zhu Y. A modified fangji huangqi decoction ameliorates pulmonary artery hypertension via phosphatidylinositide 3-kinases/protein kinase B-mediated regulation of proliferation and apoptosis of smooth muscle cells *in vitro* and *in vivo*. J Ethnopharmacol. (2023) 314:116544. 10.1016/j.jep.2023.116544 37088239

[8] Wang R Ma TM Liu F Gao HQ. Research progress on pharmacological action and clinical application of Stephania tetrandrae radix. Zhongguo Zhong Yao Za Zhi. (2017) 42(4):634–9. 10.19540/j.cnki.cjcmm.20170121.024 28959829

[9] Mingde Z. Observation of modified fangji huangqi decoction in the treatment of cardiogenic edema. Cardiovasc Dis Electron J Integr Trad Chin Western Med. (2016) 4(33):142.

[10] Cao J Zhang YG Xu LW Wang WP Hu YB Wu NN. Clinical study on the treatment of edema caused by heart failure by fFangji Huangqi decoction. Med Innov China. (2018) 15(16):072–5.

[11] General Assembly of the World Medical Association. World medical association declaration of Helsinki: ethical principles for medical research involving human subjects. J Am Coll Dent. (2014) 81(3):14–8. 25951678

[12] Idänpään-Heikkilä JE. WHO Guidelines for good clinical practice (GCP) for trials on pharmaceutical products: responsibilities of the investigator. Ann Med. (1994) 26(2):89–94. 10.3109/07853899409147334 8024733

[13] Chan AW Tetzlaff JM Gøtzsche PC Altman DG Mann H Berlin JA. SPIRIT 2013 Explanation and elaboration: guidance for protocols of clinical trials. Br Med J. (2013) 346:e7586. 10.1136/bmj.e7586 23303884 PMC3541470

[14] Cheng CW Wu TX Shang HC Li YP Altman DG Moher D. CONSORT Extension for Chinese herbal medicine formulas 2017: recommendations, explanation, and elaboration (traditional Chinese version). Ann Intern Med. (2017) 167(2):W7–w20. 10.7326/IsTranslatedFrom_M17-2977_1 28654988

[15] Heart failure group of Chinese society of cardiology of Chinese medical association, Chinese heart failure association of Chinese medical doctor association, editorial board of Chinese journal of cardiology. Chinese guidelines for the diagnosis and treatment of heart failure 2018. Chin J Cardiol. (2018) 10:760–89. 10.3760/cma.j.issn.0253-3758.2018.10.004 30369168

[16] McDonagh TA Metra M Adamo M Gardner RS Baumbach A Böhm M. 2021 ESC guidelines for the diagnosis and treatment of acute and chronic heart failure. Eur Heart J. (2021) 42(36):3599–726. 10.1093/eurheartj/ehab368 34447992

[17] Pfeffer MA Shah AM Borlaug BA. Heart failure with preserved ejection fraction in perspective. Circ Res. (2019) 124(11):1598–617. 10.1161/CIRCRESAHA.119.313572 31120821 PMC6534165

[18] Fang XJ Qian BQ Wei LP Zhao Q. Clinical research of fangji huangqi decoction in treating heart failure with preserved ejection fraction. Liaoning J Trad Chine Med. (2018) 45(12):2591–3.

[19] Liyan X Weitao C Dawei Z. The effect of fangji huanggi decoction combined with dan shen decoction on kidney bloodflow and cardiac function in patients with chronic renal failure and heart failure of qi deficiencyin spleen and kidney-type. J Changchun Univ Chin Med. (2024) 40(07):775–8.

[20] Jilin F Tingting Z Shiliang Z. Potential mechanism of fangji huangqi decoction in treating chronic heart failure based on network pharmacology. J Shandong Univ Trad Chin Med. (2021) 45(03):340–6.

